# Recurrent Vestibular Symptoms Not Otherwise Specified: Clinical Characteristics Compared With Vestibular Migraine and Menière's Disease

**DOI:** 10.3389/fneur.2021.674092

**Published:** 2021-06-17

**Authors:** Julia Dlugaiczyk, Thomas Lempert, Jose Antonio Lopez-Escamez, Roberto Teggi, Michael von Brevern, Alexandre Bisdorff

**Affiliations:** ^1^Department of Otorhinolaryngology, Head and Neck Surgery, University Hospital Zurich, University of Zurich, Zurich, Switzerland; ^2^Department of Neurology, Schlosspark-Klinik, Berlin, Germany; ^3^Otology and Neurotology Group CTS 495, Department of Genomic Medicine, Centre for Genomic and Oncological Research (GENyO) Pfizer-Universidad de Granada-Junta de Andalucía, Granada, Spain; ^4^ENT Department, San Raffaele Scientific Institute, “Vita e Salute” University, Milan, Italy; ^5^Private Practice of Neurology and Department of Neurology, Charité, Berlin, Germany; ^6^Clinique du Vertige, Centre Hospitalier Emile Mayrisch, Esch-sur-Alzette, Luxembourg

**Keywords:** recurrent vestibular symptoms not otherwise specified, benign recurrent vertigo, Menière's disease, vestibular migraine, Bárány Vestibular Symptoms grid, episodic vestibular syndrome

## Abstract

Despite the huge progress in the definition and classification of vestibular disorders within the last decade, there are still patients whose recurrent vestibular symptoms cannot be attributed to any of the recognized episodic vestibular syndromes, such as Menière's disease (MD), vestibular migraine (VM), benign paroxysmal positional vertigo (BPPV), vestibular paroxysmia, orthostatic vertigo or transient ischemic attack (TIA). The aim of the present international, multi-center, cross-sectional study was to systematically characterize the clinical picture of recurrent vestibular symptoms not otherwise specified (RVS-NOS) and to compare it to MD and VM. Thirty-five patients with RVS-NOS, 150 patients with VM or probable VM and 119 patients with MD were included in the study. The symptoms of RVS-NOS had been present for 5.4 years on average before inclusion, similar to VM and MD in this study, suggesting that RVS-NOS is not a transitory state before converting into another diagnosis. Overall, the profile of RVS-NOS vestibular symptoms was more similar to VM than MD. In particular, the spectrum of vestibular symptom types was larger in VM and RVS-NOS than in MD, both at group comparison and the individual level. However, in contrast to VM, no female preponderance was observed for RVS-NOS. Positional, head-motion and orthostatic vertigo were reported more frequently by patients with RVS-NOS than MD, while external vertigo was more prevalent in the MD group. At group level, the spectrum of attack durations from minutes to 3 days was evenly distributed for VM, while a small peak for short and long attacks in RVS-NOS and a big single peak of hours in MD were discernible. In general, vertigo attacks and associated vegetative symptoms (nausea and vomiting) were milder in RVS-NOS than in the other two disorders. Some patients with RVS-NOS described accompanying auditory symptoms (tinnitus: 2.9%, aural fullness and hearing loss: 5.7% each), migrainous symptoms (photophobia, phonophobia or visual aura in 5.7% each) or non-migrainous headaches (14%), but did not fulfill the diagnostic criteria for MD or VM. Absence of a life time diagnosis of migraine headache and attack duration of <5 min were further reasons not to qualify for VM. In some RVS-NOS patients with accompanying ear symptoms, attack durations of <20 min excluded them from being diagnosed with MD. These findings suggest that RVS-NOS is a stable diagnosis over time whose overall clinical presentation is more similar to VM than to MD. It is more likely to be composed of several disorders including a spectrum of mild or incomplete variants of known vestibular disorders, such as VM and MD, rather than a single disease entity with distinct pathognomonic features.

## Introduction

The Bárány Society began to develop the International Classification of Vestibular Disorders (ICVD) in 2006 (1). Following a systematic categorization of vestibular symptoms (2), diagnostic criteria for the most common episodic vestibular disorders were published, including vestibular migraine (VM) (3), Menière's disease (MD) (4), benign paroxysmal positional vertigo (BPPV) (5), vestibular paroxysmia (VP) (6) and hemodynamic orthostatic dizziness/vertigo (7). Despite this huge progress in international standardization, there are still quite a number of patients whose episodic vestibular symptoms cannot be explained by these or other vestibular disorders (including, but not limited to, third-window syndromes, episodic ataxia, vertebrobasilar TIAs). These recurrent vestibular symptoms of unknown etiology are usually referred to as benign recurrent vertigo (BRV) or recurrent vestibulopathy (RV) (8, 9) for adult patients. Recently, the term “recurrent vertigo of childhood” has been defined for children who do not fulfill the criteria for “vestibular migraine of childhood” (10).

Originally, BRV and RV were defined as recurrent episodes of acute-onset vertigo without cochlear symptoms or signs and not accompanied by other neurological symptoms (8, 9). Slater (8) described the duration of the core event between 1 min and 24 h, often followed by a period of positional vertigo for hours to days. These criteria were modified in subsequent studies. While some authors defined attack duration between “minutes to hours” (11–13), others requested a duration between 5 min to 24 or 72 h (9, 14, 15).

Likewise, symptom quality was defined differently. Some authors (8, 12, 16) excluded patients with head-motion triggered vertigo. Brantberg and Baloh (16), van Esch (13) and van Leeuwen (15) included only patients with spontaneous vertigo, while Pan (17) chose a broader definition of “vestibular symptoms of moderate or severe intensity” not necessarily occurring spontaneously.

Furthermore, the diagnostic value of accompanying symptoms is still an issue of debate. While the original definitions excluded additional cochlear symptoms, some studies showed that around 10 to 26% of patients with recurrent vestibular symptoms of unknown cause report unilateral audiological symptoms associated with an attack (13, 16). Likewise, the role of headaches in the definition of BRV/RV has changed over the years. Slater (8) proposed a link between BRV and migraine, while Brantberg and Baloh (16) distinguished between BRV with and without migraine. With the classification of VM as a separate vestibular disorder, migraine headaches during an attack or a history of migraine became exclusion criteria for BRV/RV (15). Still, around 20% of patients with BRV / RV report non-migrainous headache as an accompanying symptom of vertigo attacks (13, 17).

It is still unknown whether recurrent vestibular symptoms not otherwise specified (RVS-NOS) that do not fulfill any of the criteria of so far established entities are part of the spectrum of established disorders, or a single disease entity with distinct pathognomonic features, or a heterogeneous group of different disorders (18). Since the existing diagnostic criteria for vestibular disorders rely mainly on clinical presentation, the aim of the present study was to systematically categorize *all* vestibular symptoms in patients with RVS-NOS using the Bárány Vestibular Symptoms grid. In line with previously defined diagnostic criteria for vestibular syndromes, we also analyzed symptom intensity, accompanying symptoms during attacks and the temporal profile of the attacks to determine whether there are specific features that help to distinguish RVS-NOS from other diagnoses, mainly MD and VM.

## Patients and Methods

### Patients

The data of the present study was collected in the multi-center, cross-sectional “Vertigo PEVS” study (PEVS = Prospective study on the phenotype of episodic vestibular syndromes) that was performed in six clinical European centers (Luxemburg, Germany, Italy, Spain) between August 2013 and March 2014. Part of the data from VM and MD patients has been published before (19). All patients were interviewed by experienced neuro-otologists (three neurologists and three otolaryngologists) with at least 12 years of clinical practice. The centers were tertiary referral outpatient clinics or vertigo clinics in general hospitals.

In total, 423 patients with an episodic vestibular syndrome were included into the Vertigo PEVS study. For the present paper, detailed analyses were performed for patients with VM, MD and RVS-NOS. The VM group comprised a total of 150 patients suffering from VM (n = 84) or probable VM (pVM, *n* = 66) according to the classification criteria of the Bárány Society (3). One hundred and nineteen (*n* = 119) patients fulfilled the diagnostic criteria for definite MD according to the American Academy of Otolaryngology—Head and Neck Surgery (AAO-HNS) (20). The AAO-HNS classification for MD was employed as patients were recruited prior to publication of the Bárány Society criteria for MD in 2015 (4). RVS-NOS (*n* = 35) was defined as an episodic vestibular syndrome with at least two episodes of vestibular symptoms according to the Bárány Vestibular Symptoms grid (2) that could not better be explained by another vestibular disorder (including, but not limited to BPPV, VP, MD, VM, vertebrobasilar TIAs or third-window syndromes). Patients fulfilling the criteria for more than one episodic vestibular disorder were excluded from this study, for example, patients diagnosed with MD and VM according to the above mentioned criteria (Supplementary Material 1, p. 11).

This study was approved by the local ethics committees of all participating centers. All patients gave written informed consent before entering the study.

### Methods

A structured questionnaire was designed to characterize patients' symptoms according to the Bárány Vestibular Symptoms grid (2) (Supplementary Material 1). This questionnaire collected all vestibular symptoms reported by patients (vertigo, dizziness, vestibulo-visual symptoms, postural symptoms), the frequency and duration of the attacks and the intensity of the symptoms. Attack duration was defined as a distinct lapse of time during which vestibular symptoms were either continuously present or in case of triggered symptoms, attack duration comprised the time interval during which a specific trigger (e.g., head motion) was able to provoke vestibular symptoms (Supplementary Material 1). It also included basic demographic data (age and gender), patient's age at onset of vestibular symptoms and a set of questions to determine the accompanying symptoms occurring during the attacks, that is, vision-related symptoms (photophobia, visual aura, diplopia), hearing-related symptoms (phonophobia, tinnitus, fullness of ear, hearing loss), vegetative symptoms (nausea, vomiting, palpitations, choking), emotional symptoms (anxiety) and headache. Patients were able to choose if accompanying symptoms occurred never, sometimes (<50% of attacks) or mostly (≥50% of attacks) (19).

To characterize the type of headache during the attack, patients were asked to indicate whether headaches were never, sometimes or mostly “hemicranial,” “pulsating,” “worse on effort” or of “moderate or severe intensity.” If patients reported at least two of these features during most of the attacks, the headache was classified as “migraine-type” (19).

In addition to answering the PEVS questionnaire, all patients underwent history taking and a clinical neurotological examination by one of the investigators including, but not limited to, testing for spontaneous, head-shaking and positional nystagmus, smooth pursuit, saccades and head impulse test. A pure tone audiogram was performed in all patients to determine bone and air conduction hearing thresholds. Additional tests to exclude other differential diagnoses were performed at the discretion of each clinician to establish the diagnosis.

Data were entered into an Excel spreadsheet (Microsoft Office 365) and analyzed with GraphPad Prism software (version 9.0.0). For continuous variables (age, age at onset, disease duration, number of different vestibular symptoms or attack durations per patient), one-way ANOVA with Tukey's multiple comparison test was employed. If standard deviations were significantly different between groups, Brown-Forsythe's and Welch's ANOVA with Dunnett's T3 multiple comparisons test were used instead (21). A *p*-value < 0.05 was set to indicate significance. The remaining variables (e.g., relative frequencies of specific symptoms and attack durations) were categorical and analyzed with a Chi square test for three rows (VM, MD, and RVS-NOS). In case of a significant difference (*p* < 0.05), the two-sided Fisher's exact test including the Odds ratio (OR) with 95% confidence interval (95% CI) was performed for VM vs. RVS-NOS, VM vs. MD and RVS-NOS vs. MD. To correct for multiple (i.e., three) comparisons, the adjusted *p*-value was set to 0.017 for all variables except for attack duration, where p was corrected for 21 possible comparisons (*p* < 0.002). An OR >1 and a 95% CI not including 1 were used as indicators for a correlation between a variable and a specific disorder.

## Results

All results are listed in Supplementary Material 2. The main focus of this study was laid on features distinguishing RVS-NOS from either VM, MD or both (Table 1). In addition, differences between VM and MD were analyzed (Table 2).

**Table 1:** Categorical variables discriminating recurrent vestibular symptoms not otherwise specified (RVS-NOS) from Menière's disease (MD, Tables 1A,B), vestibular migraine (VM, Table 1C) or both (Table 1D).

**Variable**	**MD**	**RVS-NOS**	***P*-value**	**OR**	**95% CI**
**(A) Variables occurring more frequently in RVS-NOS than MD**					
Head-motion vertigo (non-spinning) (1.2.2.2)	0%	14%	0.0005	∞	5.41 to ∞
Positional vertigo (transient, that is, < 1 min, spinning) (1.2.1.1.1)	0%	11%	0.0023	∞	3.51 to ∞
Orthostatic vertigo (non-spinning) (1.2.6.2)	1%	14%	0.0024	19.67	2.46 to 233.1
Mostly mild attacks	3%	23%	0.0009	8.52	2.64 to 26.44
Positional vertigo (persistent, i.e., >1 min, spinning) (1.2.1.2.1)	3%	17%	0.0047	8.00	2.10 to 30.02
Orthostatic vertigo (spinning) (1.2.6.1)	3%	20%	0.003	7.19	2.03 to 22.78
Palpitations	3%	17%	0.0097	5.95	1.60 to 19.36
Head-motion vertigo (spinning) (1.2.2.1)	8%	29%	0.0023	4.89	1.89 to 12.78
**(B) Variables occurring more frequently in MD than RVS-NOS**					
Attack duration 1–4 h	66%	17%	< 0.0001	9.20	3.46 to 22.97
Nausea	81%	34%	< 0.0001	8.00	3.42 to 17.39
Vomiting	46%	11%	0.001	6.67	2.31 to 18.29
Occurrence of severe attacks in patients with mostly mild or moderate attacks	78%	35%	0.027	6.42	1.82 to 19.42
Headache (any type)	41%	14%	0.0043	4.2	1.61 to 10.48
External vertigo (3.1)	59%	26%	0.0009	4.13	1.80 to 10.06
Occurrence of attacks in clusters	59%	34%	0.0125	2.74	1.27 to 6.16
**(C) Variables occurring more frequently in VM than RVS-NOS**					
Headache (any type)	82%	14%	< 0.0001	27.3	10.0 to 68.04
Occurrence of severe attacks in patients with mostly mild or moderate attacks	83%	35%	0.0002	8.80	2.88 to 29.32
Proportion of female patients	85%	51%	< 0.0001	5.50	2.35 to 11.86
Nausea	61%	34%	0.0045	3.04	1.45 to 6.64
**(D) Variables distinguishing RVS-NOS from VM** *and* **MD (*****p*** **< ** **0.017 each)**					
**Variable**	**VM**	**MD**	**RVS-NOS**		
Headache (any type)	82%	41%	14%		
Occurrence of severe attacks in patients with mostly mild or moderate attacks	83%	78%	35%		
Nausea	61%	81%	34%		

*Only those variables occurring at significantly different frequencies between the groups were included (see section Methods for corrected p-values). All variables are listed by descending Odds ratio (OR) and 95% confidence intervals (95% CI). Index numbers of the symptoms according to the Bárány Vestibular Symptoms grid are given in brackets (see Supplementary Material 1 for an overview of all index numbers)*.

**Table 2:** Categorical variables discriminating Menière's disease (MD) from vestibular migraine (VM).

**Variable**	**VM**	**MD**	***P*-value**	**OR**	**95% CI**
**(A) Variables occurring more frequently in VM than MD**					
Head-motion vertigo (non-spinning) (1.2.2.2)	12%	0%	< 0.0001	∞	4.42 to ∞
Positional vertigo (transient, i.e., < 1 min, spinning) (1.2.1.1.1)	12%	0%	< 0.0001	∞	4.42 to ∞
Visually induced vertigo (non-spinning) (1.2.3.2)	11%	0%	< 0.0001	∞	3.79 to ∞
Positional vertigo (persistent, i.e., >1 min, non-spinning) (1.2.1.2.2)	8%	0%	0.0007	∞	2.87 to ∞
Positional vertigo (persistent, i.e., >1 min, spinning) (1.2.1.2.1)	29%	3%	< 0.0001	15.54	5.15 to 48.7
Positional dizziness (persistent) (2.2.1.2)	9%	1%	0.0042	11.2	1.76 to 120.4
Orthostatic vertigo (non-spinning) (1.2.6.2)	8%	1%	0.0077	10.26	1.56 to 110.9
Headache (any type)	82%	41%	< 0.0001	6.51	3.69 to 11.29
Oscillopsia (head-movement dependent) (3.2.1)	9%	2%	0.0086	6.02	1.56 to 27.02
Orthostatic vertigo (spinning) (1.2.6.1)	17%	3%	0.0005	5.75	2.10 to 15.67
Proportion of female patients	85%	55%	< 0.0001	4.83	2.71 to 8.48
Palpitations	14%	3%	0.0027	4.68	1.62 to 12.89
Spontaneous vertigo (non-spinning) (1.1.2)	25%	8%	0.0003	3.70	1.75 to 7.48
Head-motion vertigo (spinning) (1.2.2.1)	23%	8%	0.0007	3.58	1.70 to 7.90
Visually induced dizziness (2.2.3)	18%	6%	0.0029	3.51	1.54 to 8.85
Orthostatic dizziness (2.2.6)	17%	6%	0.0047	3.36	1.46 to 8.49
Head-motion dizziness (2.2.2)	22%	8%	0.0025	3.07	1.50 to 6.24
Exacerbations within attacks	49%	25%	< 0.0001	2.81	1.68 to 4.76
Movement-induced blur (3.5)	21%	10%	0.0134	2.42	1.18 to 4.80
Spontaneous dizziness (2.1)	37%	18%	0.0006	2.27	1.53 to 4.75
**(B) Variables occurring more frequently in MD than VM**					
Attack duration 1–4 h	30%	66%	< 0.0001	4.44	2.61 to 7.32
Vomiting	17%	46%	< 0.0001	4.30	2.49 to 7.58
Nausea	61%	81%	0.0008	2.63	1.53 to 4.69
External vertigo (3.1)	37%	59%	0.0006	2.40	1.46 to 3.89
Clusters lasting months	12%	33%	0.004	3.73	1.49 to 8.75

*Only those variables occurring at significantly different frequencies between the groups were included (see Methods section for corrected p-values). All variables are listed by descending Odds ratio (OR) and 95% confidence intervals (95% CI). Index numbers of the symptoms according to the Bárány Vestibular Symptoms grid are given in brackets (see Supplementary Material 1 for an overview of all index numbers)*.

### Age and Gender

The mean age at onset of symptoms was younger for VM (42.43 ± 13.58 years) than for MD (48 ± 13.14 years; ANOVA with Tukey's test for multiple comparisons: *p* = 0.0019). No statistically significant difference was observed for patients with RVS-NOS (47.16 ± 14.24 years) as compared to the other two groups (VM: *p* = 0.15; MD: *p* = 0.92). The mean disease duration on inclusion into the study was >5 years for all three groups. MD patients had a longer history of recurrent vestibular symptoms than those with VM (7.63 ± 8.09 years vs. 5.26 ± 6.59 years; *p* = 0.02), while there was no significant difference between RVS-NOS patients (5.40 ± 6.10 years) and the other two groups (VM: *p* = 0.99; MD: *p* = 0.24).

The proportion of female patients was higher in VM (85%) than in MD (55%) and RVS-NOS (51%; two-sided Fisher's exact test corrected for multiple comparisons: *p* < 0.0001 each; Tables 1C, 2A).

### Bárány Vestibular Symptoms Grid

#### RVS-NOS vs. VM and MD

**Figure 1:**
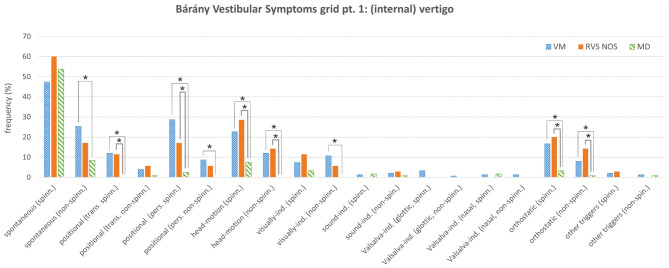
Bárány Vestibular Symptoms grid, part 1: (internal) vertigo. Relative frequencies (%) of the different symptoms are depicted for vestibular migraine (VM), recurrent vestibular symptoms not otherwise specified (RVS-NOS) and Menière's disease (MD). Multiple answers were possible. Symptoms that occurred with significantly different frequencies between groups are marked with an asterisk (*). ind., induced; pers., persistent (≥1 min); spinn., spinning; trans., transient (< 1 min).

**Figure 2:**
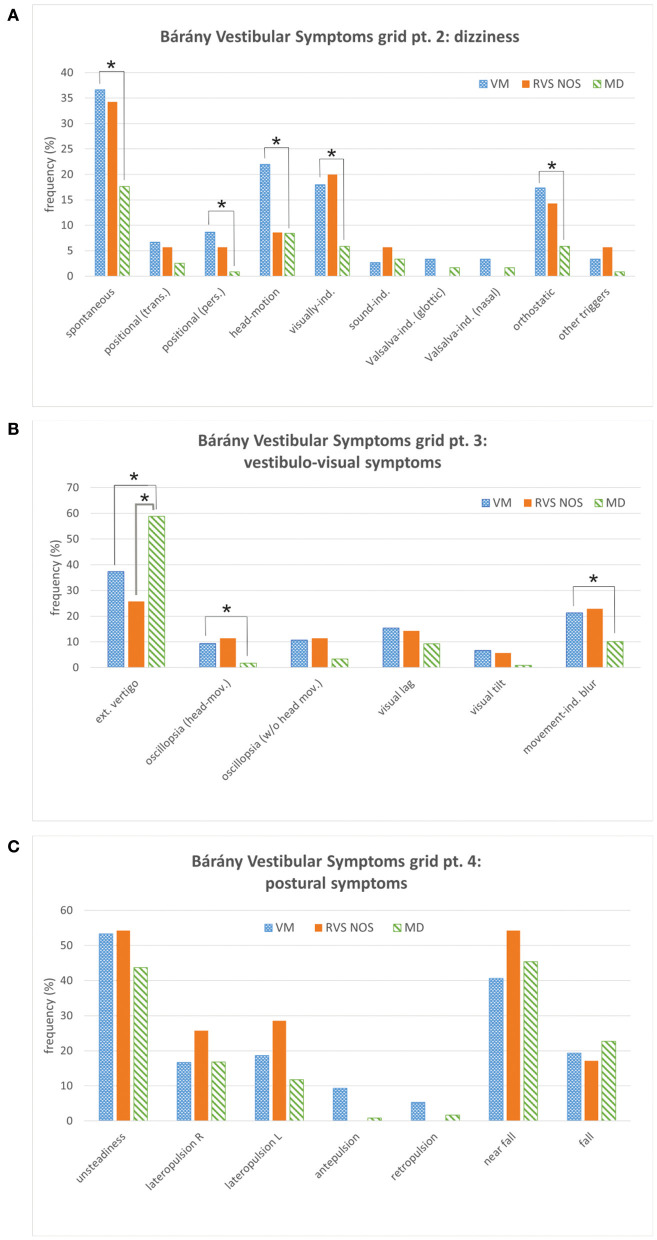
Bárány Vestibular Symptoms grid, parts 2 to 4. **(A)** Dizziness. **(B)** Vestibulo-visual symptoms. **(C)** Postural symptoms. See Figure 1 for details. Multiple answers were possible. Symptoms that occurred with significantly different frequencies between groups are marked with an asterisk (*). ext., external; ind., induced; L, left; mov., movement; pers., persistent (≥1 min); R, right; trans., transient (< 1 min).

In general, the spectrum of vestibular symptoms according to the Bárány Vestibular Symptoms grid was broader in RVS-NOS and VM than in MD. First, the average number of different vestibular symptoms per patient was higher in VM and RVS-NOS than in MD. A patient with VM reported 6.03 ± 4.02 (mean ± SD) different vestibular symptoms (median: 5), which was not statistically different from the 5.91 ± 2.81 symptoms (median: 5) experienced by an RVS-NOS patient (two-sided Fisher's exact test: *p* = 0.99). On the other hand, only 3.61 ± 2.80 symptoms (median: 2) were described on average per patient with MD, which was significantly less compared to both VM (*p* < 0.0001) and RVS-NOS (*p* = 0.0002). Second, the type of vestibular symptoms on the group level was very similar for patients with RVS-NOS and VM. None of the symptoms from the Bárány Vestibular Symptoms grid occurred at statistically different frequencies between these two groups (Figures 1, 2 and Table 1C). On the other hand, some symptoms were reported less frequently by patients with MD than those with RVS-NOS (Table 1A) and VM (Table 2A).

Regarding internal vertigo (part one of the Symptoms grid), positional, head-motion triggered and orthostatic vertigo were experienced more often by patients with RVS-NOS than those with MD (Table 1A and Figure 1). Non-spinning head-motion triggered vertigo and transient spinning positional vertigo were only reported by RVS-NOS (14 and 11% each), but not by MD patients. Spontaneous spinning vertigo was the most common vestibular symptom in all three disorders occurring at relative frequencies of 60% in RVS-NOS, 54% in MD and 47% in VM (Supplementary Material 2). There was, however, no significant difference between the groups (Chi-square test of three rows: *p* = 0.32).

The symptom dizziness (Symptoms grid, part 2) provided no additional information for the discrimination between RVS-NOS and MD (Figure 2A). External vertigo (i.e., the false sensation that the visual surround is spinning or flowing) was the only vestibulo-visual symptom (Symptoms grid, part 3) distinguishing RVS-NOS from MD, and the only vestibular symptom that occurred more often in MD (59%) than in RVS-NOS (26%) and VM (37%) (Figure 2B and Tables 1B, 2B). None of the postural symptoms (Symptoms grid, part 4) occurred with significantly different frequencies between the groups (Figure 2C).

#### VM vs. MD

As mentioned above, many symptoms of the Bárány Vestibular Symptoms grid, part 1–3, were reported at different frequencies by patients with VM compared to those with MD (Table 2 and Figures 1, 2). In line with the broader spectrum of symptoms in VM as compared to MD, almost all vestibular symptoms occurred more often in VM than in MD (Table 2A), apart from external vertigo, which was more common in MD (Table 2B). Of note, there were some symptoms of internal vertigo that were only described by patients with VM (non-spinning head-motion vertigo, positional vertigo and visually induced vertigo), but not by those with MD. Postural symptoms (Symptoms grid, part 4) were not significantly different between VM and MD (Figure 2C).

### Accompanying Symptoms During Attacks

**Figure 3:**
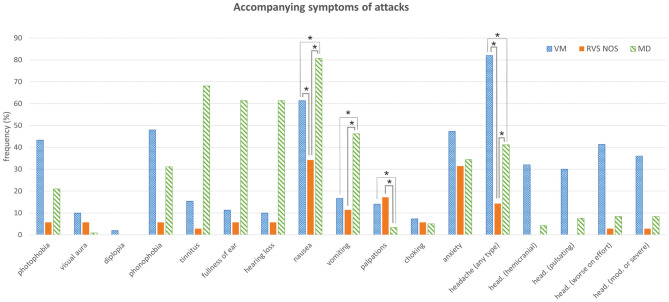
Accompanying symptoms of attacks. See Figure 1 for details. Multiple answers were possible. Symptoms that occurred with significantly different frequencies between groups are marked with an asterisk (*). head., headache; mod., moderate.

Only accompanying symptoms that are not an integral part of a disease definition were analyzed in the three groups in order to avoid circular argumentation. The only two accompanying symptoms occurring at significantly different frequencies during most attacks (i.e., >50%) in all three groups (Figure 3 and Tables 1, 2) were nausea (MD: 81%, VM: 61%, RVS-NOS: 34%) and headache of any type (VM: 82%, MD: 41%, RVS-NOS: 14%). In addition, vomiting was more common in MD (46%) than in VM (17%) or RVS-NOS (11%), while palpitations were reported less frequently by MD patients (3%) as compared to the two other disorders (VM: 14%, RVS-NOS: 17%).

Most of the patients did not receive a diagnosis of VM or MD because they did not exhibit any pathognomonic accompanying symptoms (Table 3). Headache was experienced by 14% of RVS-NOS patients, however, none of them displayed the characteristic features of migraine-type headaches (see section Methods), indicating that the ICVD criteria were correctly applied during the study. There was a small fraction of RVS-NOS patients with other migrainous symptoms during attacks (14.3% in total; photophobia or visual aura or phonophobia in 5.7% (*n* = 2) each, multiple answers possible; Figure 3). These patients did not fulfill the diagnostic criteria for pVM/VM, either because they reported only phonophobia (*n* = 2) or only photophobia (*n* = 2) or because attack duration was < 5 min (*n* = 2 patients with visual aura). None of the RVS-NOS patients reported a migraine history, thus excluding pVM. Accompanying auditory symptoms were reported by 8.6% of RVS-NOS patients (tinnitus in 2.9%, fullness of ear and hearing loss in 5.7% each; multiple answers possible). These patients were not diagnosed with MD because attack duration was always < 20 min.

**Table 3:** Reasons why RVS-NOS patients were not diagnosed with vestibular migraine (VM) or Menière's disease (MD) based on accompanying symptoms and duration of attacks.

**Variable**	**RVS-NOS patients (%)**
**Vestibular migraine**	
No accompanying photophobia, phonophobia and/or visual aura	85.7%
No accompanying migraine-type headache	100%
Attack duration < 5 min	48.6%
**Menière's disease**	
No accompanying auditory symptoms (hearing loss, tinnitus and/or fullness of ear)	91.4%
Attack duration < 20 min	48.6%

### Intensity of Attacks

Patients were asked whether they would rate most of their attacks as mild (does not interfere with daily activities), moderate (interferes with daily activities) or severe (daily activities not possible). Those who reported mostly mild or moderate attacks were asked whether they also experienced severe attacks.

Mostly mild attacks were reported more frequently by patients with RVS-NOS (23%) than MD (3%; two-sided Fisher's exact test: *p* = 0.0009) (Table 1A). The occurrence of severe attacks in those patients with mostly mild or moderate attacks was more frequent in patients with VM (83%) and MD (78%) than those with RVS-NOS (35%; see Tables 1B–D for statistical analysis). Both observations indicate a less severe attack intensity in RVS-NOS compared to the other two disorders.

Summarizing sections Age and Gender, Bárány Vestibular Symptoms Grid, Accompanying Symptoms During Attacks, and Intensity of Attacks, there were only three features that distinguished patients with RVS-NOS from *both* the MD and the VM groups: less headache of any type, less nausea, and less occurrence of severe attacks in patients with mostly mild or moderate attacks (Table 1D).

### Temporal Characteristics of Attacks

#### Attack Frequency and Duration

**Figure 4:**
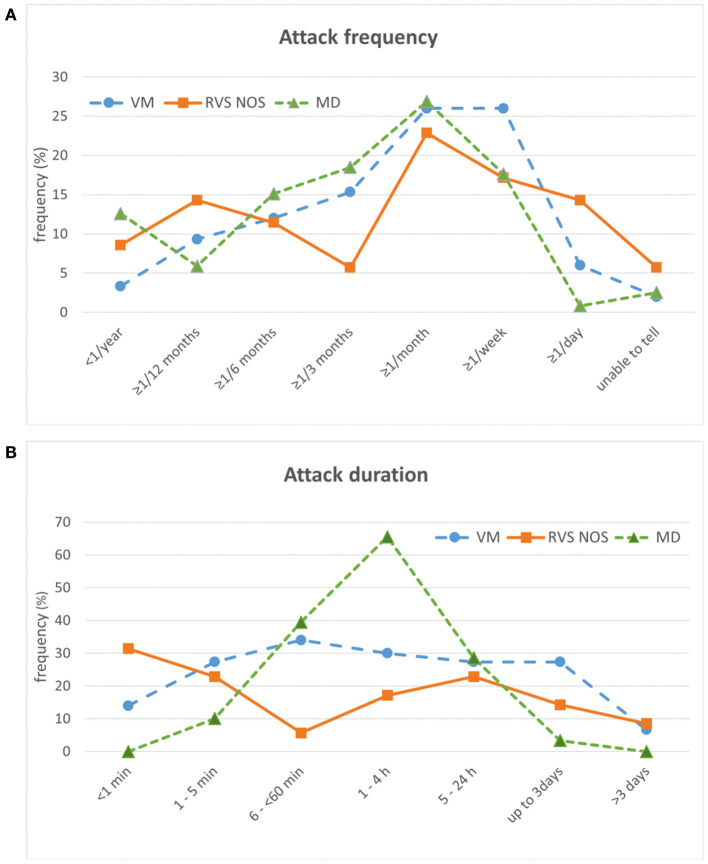
Temporal characteristics of attacks in patients with vestibular migraine (VM), recurrent vestibular symptoms not otherwise specified (RVS-NOS) and Menière's disease (MD) (relative frequencies in %). **(A)** Attack frequency. Only one answer was possible. **(B)** Attack duration. Multiple answers were possible.

Regarding attack frequency, patients had to choose one answer in the “Vertigo PEVS” questionnaire (Supplementary Material 1 and section Rationale of the Present Study). For all three disorders, the most common attack frequency was “≥ 1/month” (i.e., at least one attack per month, but < 1 per week), reported by 27% (MD), 26% (VM), and 23% (RVS-NOS) of patients each (Figure 4A). While MD patients displayed a single peak for this attack frequency, the distribution was more even in VM, where another 26% chose “≥ 1/week” as most common attack frequency (i.e., at least one attack per week, but < 1 per day). A second peak at “≥ 1/year” (i.e., at least one attack per year, but < 1 attack within 6 months) was observed for 14% of RVS-NOS patients.

For attack duration, multiple answers were possible (see question 5.1 in Supplementary Material 1). On the group level, MD patients displayed a single peak (66% of patients) with an attack duration from one to 4 h (Figure 4B). This duration allowed to distinguish patients with MD from VM (OR = 4.4) and RVS-NOS (OR = 9) on the group level (see Tables 1B, 2B for further details). As observed for symptom quality above, the distribution of attack durations was more even across the spectrum of durations for VM and RVS-NOS than for MD. In VM, attack durations ranging from “1–5 min” to “up to 3 days” were evenly distributed, reported by around 30% of patients each. Two peaks were discernible for RVS-NOS (“ < 1 min”: 31% and “5–24 h”: 23%).

As more than one answer was possible for attack duration in the “Vertigo PEVS” questionnaire, we sought to identify whether the broad spectrum of attack durations observed for VM and RVS-NOS as groups was also present in the individual patients. On average, patients of all three groups reported between one and two different attack durations (VM: 1.66 ± 1.00; MD: 1.50 ± 0.62; RVS-NOS: 1.22 ± 0.48), and only 16% (VM), 6% (MD) and 3% (RVS-NOS) of patients each described more than two distinct attack durations. Thus, the majority of patients in all three groups have one or two dominant attack durations contrasting with the broad spectrum at the group level for VM and RVS-NOS.

#### Clusters of Attacks

Periods with many attacks (“clusters”) were more common in the MD (59%) than in the RVS-NOS group (34%; two-sided Fisher's exact test: *p* = 0.0125) (Table 1B). Clusters lasting for months (instead of weeks) were more often reported by patients with MD (33%) than those with VM (12%, two-sided Fisher's exact test: *p* = 0.004), while no significant difference was observed between patients with RVS-NOS (8%) and the other two disorders (Table 2B).

## Discussion

### Rationale of the Present Study

Most of the previous definitions of RV/BRV put restrictions on either the quality of vestibular symptoms (only spontaneous attacks of vertigo not precipitated by head movements) or their duration (5 min to 24 or 72 hours) and excluded patients with any accompanying auditory or neurological symptoms (8, 9, 13, 15, 16, 22, 23). On the other hand, studies performed before publication of the diagnostic criteria of VM (3) often did not exclude patients with a history of migraine headache and/or accompanying migrainous symptoms during a vertigo attack (22–24), and many of the cases classified as BRV then, for example, (25), would be diagnosed with VM according to the ICVD criteria today. Thus, the results of these earlier works may be biased by the inclusion of patients considered to have VM according to present criteria.

Here, we decided to classify all those patients as RVS-NOS, whose recurrent vestibular symptoms were not better accounted for by any other recognized episodic vestibular disorder—including VM—regardless of the type of vestibular symptoms, duration of the attacks and presence of accompanying symptoms. We chose the term “recurrent vestibular symptoms not otherwise specified” (RVS-NOS) for these patients in order to indicate that inclusion criteria were different as compared to previous studies. To the best of our knowledge, this is the first international, multi-center study applying the Bárány Vestibular Symptoms grid to a large number of patients with RVS-NOS, MD and VM (305 in total). The aim of this approach was to provide a “real life” picture of those episodic vestibular syndromes that are not captured by any of the current vestibular disease criteria.

### Characteristic Features of RVS-NOS

#### Vestibular Symptoms

One of the most important findings of the present study is the broad spectrum of vestibular symptoms reported by patients with RVS-NOS, both on the group and on the patient level (Figures 1, 2). Spontaneous spinning vertigo was the most common vestibular symptom in RVS-NOS patients (60%). It was however not found in every patient, thus confirming the observation by Pan et al., where spontaneous vertigo was experienced by most (77,8%), but not all patients with BRV (17). Besides spontaneous vertigo, spontaneous dizziness was commonly reported by RVS-NOS patients in our study (34%) and the study by Pan et al. (15%).

While none of the symptoms from the Bárány Vestibular Symptoms grid occurred at different frequencies in RVS-NOS vs. VM in the present study, patients with RVS-NOS experienced certain vertigo types (head-motion, positional and orthostatic) more often than those with MD (Table 1A and Figure 1). In order to capture this broad spectrum of vestibular symptoms, inclusion criteria for RVS-NOS should comprise all kinds of vestibular symptoms in contrast to previous definitions of BRV and RV that were often restricted to spontaneous (spinning) vertigo (see section Rationale of the Present Study).

Of note, positional vertigo without nystagmus or persistent positional nystagmus not compatible with BPPV have been observed in patients with BRV before (11, 24), and Slater (8) described a period of positional vertigo following the core event of a BRV attack (8). These observations from previous studies might have been contaminated by patients with VM who may present with positional vertigo / nystagmus during an attack as well (26–28). It is possible that some RVS-NOS patients with positional vertigo might actually suffer from BPPV, as the pathognomonic BPPV-type nystagmus may not be visible on every examination (5). On the other hand, diagnosis had to be changed from BRV to BPPV in only 9% of patients after a follow-up of 2 to 8.5 years in two studies (11, 14). In line with these previous observations are the data from our study where the mean disease duration was not different for RVS-NOS patients with attack durations < 1 min (4.75 ± 7.73 years), between 1 and 5 min (5.77 ± 8.80 years) and longer than 5 min (6.32 ± 5.28 years). Not to make a BPPV diagnosis over such a long period would be highly unlikely.

The vestibular syndrome of RVS-NOS seems to be stable over time rather than a transitory condition converting into another vertigo syndrome. Previous studies reported that the diagnosis of patients with BRV/RV had to be changed to migraine in only 2 to 7.5% of cases and to MD in only 1 to 4% over median follow-up times between 31 and 63 months (12, 17, 29). This is in line with the results from our study where the mean duration of the vestibular syndrome before the patients' first visit in a neurotology clinic was not significantly different between patients with RVS-NOS (5.40 ± 6.10 years) as compared to VM (5.26 ± 6.59 years) or MD (7.63 ± 8.09 years) indicating a rather stable vestibular syndrome not changing over the years.

#### Accompanying Symptoms

Accompanying auditory or migraine-type symptoms in ≥50% of attacks were observed in some RVS- NOS patients although all patients fulfilling the criteria for VM/pVM and MD were excluded from this group. 2.9% reported tinnitus, while hearing loss or aural fullness were each present in 5.7%. First, these low rates of accompanying auditory symptoms indicate that the “Vertigo PEVS” questionnaire was applied correctly by the investigators. Second, the fact that some of the patients with RVS-NOS *did* report auditory symptoms without fulfilling the criteria of MD raises the question whether exclusion of auditory symptoms should be part of the diagnostic criteria for RVS-NOS as proposed before (8, 9). Similar rates of accompanying auditory symptoms (3–14%) have been reported for BRV patients without migraine before. Furthermore, the relative frequencies of photophobia *or* phonophobia (5.7% each) and non-migrainous headache (14%) in our study were similar to values from the literature (13, 16, 17, 23).

RVS-NOS patients experienced nausea during ≥50% of attacks less frequently (34%) than patients with VM (61%) or MD (81%); vomiting was less common (11%) than in patients with MD (46%). Both findings indicate a relatively mild attack intensity in RVS-NOS as compared to the other two groups (for details see section Disease Severity). The low prevalence of vomiting in the present study is in accordance with the first description of BRV (8), where none of the patients reported vomiting associated with an attack. In general, frequencies for nausea and vomiting in the present study were lower for all three disorders compared to previous reports (11, 13). This discrepancy is most likely due to the fact that we counted only patients suffering from these symptoms during most of (i.e., ≥50%) the attacks.

No significant difference was observed between the proportion of RVS-NOS, VM and MD patients who experienced anxiety during the attacks (31, 47, and 34%, respectively). This is in line with the study by van Esch et al. (13), who found no difference in Hospital Anxiety and Depression Scores (HADS) between these patient groups (13).

#### Disease Severity

Several factors in the present study advocate that disease severity was generally milder in patients with RVS-NOS as compared to those with MD or VM. First, the fraction of patients suffering mostly from mild attacks was higher for RVS-NOS (23%) than MD patients (3%). In addition, less patients with mostly mild or moderate attacks experienced also severe attacks in RVS-NOS (35%) than in VM (83%) or MD (78%). Second, clusters of attacks occurred less frequently in the RVS-NOS (34%) as compared to the MD group (59%). Finally, the relatively mild nature of the attacks was reflected by the lower prevalence of nausea and vomiting as compared to the other two disorders (see section Accompanying Symptoms).

#### Temporal Characteristics of Attacks

At group level, RVS-NOS patients displayed a “two-peak” pattern for attack frequency and duration contrasting the “single-peak” pattern for MD and the “plateau-like” distribution for VM (Figure 4). The even distribution of attack durations on the group level for VM resembles the results from previous studies on the duration of headaches and vertigo attacks in VM (30, 31). For RVS-NOS, the second peak was always localized within the lower range of reported values in the present study, that is, attack duration < 1 min (31%) and more than one attack per year, but < 2 within 6 months (14%).

This observation has several implications. First, it confirms the notion that a subset of patients with RVS-NOS shows a relatively mild disease severity (see above). Second, the high proportion of attacks < 1 min (31%) indicates that there is a considerable number of patients with short-lived recurrent vestibular symptoms that cannot be classified as BPPV or VP, confirming observations by Pan et al. (duration < 5 min in 22.5%) and Lee et al. (duration < 10 min in 6%) (12, 17). Considering the stable course of the vestibular syndrome in these patients (see section Vestibular Symptoms), it is rather unlikely that they will convert into BPPV or VP on the long term. Third-window syndromes are also an unlikely differential diagnosis, as symptoms like sound- and pressure induced vertigo / dizziness were virtually absent in the RVS-NOS group of the present study (Figures 1, 2A).

Of note, a bimodal distribution of attack duration for BRV on the group level has been reported before by Lee et al. (“few minutes”: 38.9%; “few hours”: 51.4%) and Brantberg and Baloh (1–5 min: 20% and 1–4 h: 30%) (16, 24). In contrast to the broad spectrum of attack durations on the group level in the present study (Figure 4B), only 3% of RVS-NOS patients reported more than two different attack durations on the individual patient level. In summary, these distributions of attack duration on the group and individual level indicate that RVS-NOS is—at least currently—a heterogeneous subset of disorders that need to be further characterized in future studies. We propose that there should be no lower limit of attack duration in the definition of RVS-NOS in order to grasp the full spectrum of this multi-facetted disorder.

### RVS-NOS in Relation to Other Episodic Vestibular Syndromes

#### Vestibular Migraine

The different symptoms of the Bárány Vestibular Symptoms grid occurred with similar frequencies in patients with VM and RVS-NOS. In particular, none of these *vestibular* symptoms allowed to distinguish between the two disorders. While previous studies suggested a link between BRV/RV and migraine (8, 11, 22–25), several findings from the present study indicate that RVS-NOS is *not* just another migraine variant.

First, we observed a balanced gender distribution in patients with RVS-NOS (51% females) in contrast to the well-known female preponderance in VM of 85% in the present study (32–34), confirming the results of the study by van Esch (13). At this point, it should be noted that the female preponderance described in BRV/RV studies before 2012 (8, 23–25) might be due to inclusion of patients that would probably have been diagnosed with VM today (see also Section Rationale of the Present Study). This notion is supported by Brantberg and Baloh, who found a female preponderance only for those BRV patients with a positive migraine history (84% female), while those without migraine displayed a more balanced gender distribution (58%) (16).

Second, the temporal profile of vertigo attacks was different between RVS-NOS and VM in the present study. In summary, patients with RVS-NOS displayed two peaks in the distribution of attack frequencies and duration on the group level in contrast to the “plateau pattern” observed for patients with VM (Figure 4). Although the pathophysiological correlate for the two peaks in RVS-NOS is not clear to date (see Section Temporal Characteristics of Attacks), this observation might contribute to a better separation between the two disorders in clinical practice.

#### Menière's Disease

In general, the clinical presentation of MD patients in the present study was more stereotyped as compared to RVS-NOS: the spectrum of vestibular symptoms was narrower both on the group and on the individual patient level (Figures 1, 2). While a “double-peak” pattern was observed for attack frequency and duration on the RVS-NOS group level, MD patients displayed a single peak for both parameters (Figure 4). The marked stereotypic pattern of MD attacks might be due to a common underlying pathology in these patients, such as endolymphatic hydrops (35), which is present on inner ear hydrops MRI in almost all patients with MD (36, 37).

A further characteristic feature of MD in the present study was the relatively high proportion of patients experiencing attack clusters of several months (33%). This has an important implication for designing clinical studies with MD patients: if patients are recruited during a cluster of attacks and then return back to baseline, one may have the illusion of a treatment effect, in particular if the endpoint is defined several months or years after inclusion into the study.

It has been debated whether recurrent vertigo attacks without accompanying hearing loss are a subset of MD (“vestibular MD”) (38). In around 20% of cases, MD begins with isolated vestibular symptoms. While 80% of patients develop the full audiovestibular spectrum of symptoms within 5 years, patients with merely vestibular symptoms and without hearing loss over periods of 20 years and more have been described (36, 38). A closer look at the study by Paparella and Mancini reveals, however, that all but one of 51 patients diagnosed with vestibular MD reported aural fullness and 84% suffered from tinnitus. These accompanying symptoms were only encountered in 5.7 and 2.9% of RVS-NOS patients each in the present study. Therefore, it seems unlikely that RVS-NOS is just another subgroup of MD. Nevertheless, a possible conversion into MD cannot be excluded, in particular in those with a disease duration < 5 years (63% in the present study).

#### Possible Causes of RVS-NOS

So, what are the mechanisms behind RVS-NOS? As this is a symptom-oriented study, it can only provide a tentative answer to this question.

We were not able to identify any pathognomonic vestibular symptoms or other operational criteria that clearly separated RVS-NOS from already known vestibular disorders. Therefore, it seems unlikely that RVS-NOS represents a single, so far unrecognized disease entity. The number and the quality of vestibular symptoms in patients with RVS-NOS (both on the group and on the patient level) suggest that part of these patients suffer from a mild form of VM, while the subgroup with auditory symptoms might represent a very mild form of MD. But it probably also includes some as yet unidentified entities, like recurrent spontaneous vertigo with interictal headshaking nystagmus which was only described after our data collection (39).

In summary, these findings indicate that RVS-NOS is a heterogenous group of different disorders with relatively mild clinical presentations—too mild to fulfill the current diagnostic criteria of, for example, VM or MD- and as yet unidentified diseases, one of which was only described after our data collection.

### Limitations of the Present Study

This study has several limitations. First, the number of patients with RVS-NOS was quite small compared to those with MD and VM. Studies with larger patient numbers would be desirable in the future to explore whether the results of the present study are representative for RVS-NOS.

Second, patients were recruited before the Bárány Society classifications for MD, BPPV and VP were published. Therefore, the AAO-HNS criteria were applied to identify patients with definite MD, and a customized definition for VP was used (see Supplementary Material 1). In particular, probable MD (either according to the AAO-HNS or the Barany Society criteria) was not listed as a separate diagnosis in the PEVS questionnaire. For the purpose of the present study, the Bárány Society criteria for MD, BPPV and VP (both definite and probable) were retrospectively applied to the RVS-NOS group in order to exclude all patients whose symptoms could better be explained by another vestibular disorder.

Third, we only performed a clinical neurotological examination in order to rule out other vestibular disorders, such as BPPV. Additional vestibular tests (e.g., caloric irrigation, video head impulse testing, video-nystagmography) and imaging (e.g., hydrops imaging of the inner ear) were not part of this study. It is unclear to date, whether these examinations have an additional value in the differential diagnosis of RVS-NOS. For instance, a recent study has helped to identify a specific subset of patients with recurrent spontaneous vertigo whose head-shake nystagmus is clearly different from those of MD and VM patients indicating that the attacks are caused by hyperactivity and asymmetry in the vestibular velocity storage mechanism (39, 40).

Finally, the familial history of auditory or vestibular symptoms was not obtained in the present study. However, both VM and MD show a significant familial aggregation (41, 42), and families with either VM or MD may have individuals with partial syndromes that could fit in the diagnosis of RVS-NOS. Future studies should investigate familial aggregation of RVS-NOS and VM.

## Conclusion

The present study suggests that RVS-NOS is a heterogeneous group of vestibular disorders. The stability of symptoms over time indicates that it is most likely not a transition phase before fulfilling the criteria of other well-defined vestibular entities.

There are inherent limitations to what phenotyping of vestibular symptoms may achieve in terms of diagnosis. All vestibular symptoms are non-specific, patterns of symptoms may be more in favor of one or the other entity, but in MD and VM the accompanying symptoms of hearing loss, the audiogram and the other non-vestibular migrainous symptoms determine the diagnostic classification.

Long term follow-up, examination of patients during an attack, future use of biomarkers and possible treatment response may help to further clarify whether RVS-NOS is part of the spectrum of already defined disorders or one or more separate disorders.

## Data Availability Statement

The original contributions presented in the study are included in the article/Supplementary Material, further inquiries can be directed to the corresponding author/s.

## Ethics Statement

The studies involving human participants were reviewed and approved by Comité National d'Ethique de Recherche (National Research Ethics Committee, CNER), Luxembourg. The patients/participants provided their written informed consent to participate in this study.

## Author Contributions

JD and AB analyzed the data and wrote a first draft of the manuscript. All authors designed the study, performed clinical assessment, neurotological examination of study subjects, collected the data, contributed to manuscript revision, read, and approved the submitted version.

## Conflict of Interest

The authors declare that the research was conducted in the absence of any commercial or financial relationships that could be construed as a potential conflict of interest.

## Supplementary Material

Supplementary Material 1 “Vertigo PEVS” questionnaire (PEVS = prospective study on the phenotype of episodic vestibular syndromes).

Supplementary Material 2 Results of the “Vertigo PEVS” questionnaire for patients with vestibular migraine (VM, *n* = 150), recurrent vestibular symptoms not otherwise specified (RVS-NOS, *n* = 35) and Menière's disease (MD, *n* = 119). All results are presented as absolute numbers and relative frequencies in %.

## References

[1] Bisdorff AR Staab JP Newman-Toker DE. Overview of the international classification of vestibular disorders. Neurol Clin. (2015) 33:541–50, vii. 10.1016/j.ncl.2015.04.010 26231270

[2] Bisdorff A Von Brevern M Lempert T Newman-Toker DE. Classification of vestibular symptoms: towards an international classification of vestibular disorders. J Vestib Res. (2009) 19:1–13. 10.3233/VES-2009-0343 19893191

[3] Lempert T Olesen J Furman J Waterston J Seemungal B Carey J. Vestibular migraine: diagnostic criteria. J Vestib Res. (2012) 22:167–72. 10.3233/VES-2012-0453 23142830

[4] Lopez-Escamez JA Carey J Chung WH Goebel JA Magnusson M Mandal à M. Diagnostic criteria for Menière's disease. J Vestib Res. (2015) 25:1–7. 10.3233/VES-150549 25882471

[5] Von Brevern M Bertholon P Brandt T Fife T Imai T Nuti D. Benign paroxysmal positional vertigo: diagnostic criteria. J Vestib Res. (2015) 25:105–17. 10.3233/VES-150553 26756126

[6] Strupp M Lopez-Escamez JA Kim JS Straumann D Jen JC Carey J. Vestibular paroxysmia: diagnostic criteria. J Vestib Res. (2016) 26:409–15. 10.3233/VES-160589 28262641 PMC9249278

[7] Kim HA Bisdorff A Bronstein AM Lempert T Rossi-Izquierdo M Staab JP. Hemodynamic orthostatic dizziness/vertigo: diagnostic criteria. J Vestib Res. (2019) 29:45–56. 10.3233/VES-190655 30883381 PMC9249281

[8] Slater R. Benign recurrent vertigo. J Neurol Neurosurg Psychiatry. (1979) 42:363–7. 10.1136/jnnp.42.4.363 458483 PMC490208

[9] Leliever WC Barber HO. Recurrent vestibulopathy. Laryngoscope. (1981) 91:1–6. 10.1288/00005537-198101000-00001 6969834

[10] Van De Berg R Widdershoven J Bisdorff A Evers S Wiener-Vacher S Cushing SL. Vestibular migraine of childhood and recurrent vertigo of childhood: Diagnostic criteria consensus document of the committee for the classification of vestibular disorders of the bárány society and the international headache society. J Vestib Res. (2020) 31:1–9. 10.3233/VES-200003 33386837 PMC9249292

[11] Kentala E Pyykkö I. Benign recurrent vertigo–true or artificial diagnosis? Acta Otolaryngol Suppl. (1997) 529:101–3. 10.3109/00016489709124095 9288283

[12] Lee HK Ahn SK Jeon SY Kim JP Park JJ Hur DG. Clinical characteristics and natural course of recurrent vestibulopathy: a long-term follow-up study. Laryngoscope. (2012) 122:883–6. 10.1002/lary.23188 22374685

[13] Van Esch BF Van Wensen E Van Der Zaag-Loonen HJ Benthem P Van Leeuwen. R. B. Clinical characteristics of benign recurrent vestibulopathy: clearly distinctive from vestibular migraine and Menière's disease? Otol Neurotol. (2017) 38:e357–63. 10.1097/MAO.0000000000001553 28834943

[14] Rutka JA Barber HO. Recurrent vestibulopathy: third review. J Otolaryngol. (1986) 15:105–7. 3712538

[15] Van Leeuwen RB Bruintjes TD. Clinical features and outcomes of benign recurrent vertigo. Acta Neurol Scand. (2020) 142:83. 10.1111/ane.13241 32170722

[16] Brantberg K Baloh RW. Similarity of vertigo attacks due to Meniere's disease and benign recurrent vertigo, both with and without migraine. Acta Otolaryngol. (2011) 131:722–7. 10.3109/00016489.2011.556661 21469911

[17] Pan Q Zhang Y Zhang S Wang W Jiang H Fan Y. Clinical features and outcomes of benign recurrent vertigo: A longitudinal study. Acta Neurol Scand. (2020) 141:374–9. 10.1111/ane.13214 31883379

[18] Domínguez-Durán E Domènech-Vadillo E Bécares-Martínez C Montilla-Ibáñez MA Álvarez-Morujo De Sande MG González-Aguado R. Exploring the frontiers of vestibular migraine: a case series. J Vestib Res. (2020) 31:91–9. 10.3233/VES-201559 33361625

[19] Lopez-Escamez JA Dlugaiczyk J Jacobs J Lempert T Teggi R Von Brevern M. Accompanying symptoms overlap during attacks in Menière's disease and vestibular migraine. Front Neurol. (2014) 5:265. 10.3389/fneur.2014.00265 25566172 PMC4265699

[20] Committee on Hearing and Equilibrium. Committee on Hearing and Equilibrium guidelines for the diagnosis and evaluation of therapy in Menière's disease. American Academy of Otolaryngology-Head and Neck Foundation, Inc. Otolaryngol Head Neck Surg. (1995) 113:181–5. 10.1016/S0194-5998(95)70102-8 7675476

[21] Motulsky H. Intuitive Biostatistics. Oxford: Oxford University Press (2014).

[22] Oh AK Lee H Jen JC Corona S Jacobson KM Baloh RW. Familial benign recurrent vertigo. Am J Med Genet. (2001) 100:287–91. 10.1002/ajmg.1294 11343320

[23] Cha YH Lee H Santell LS Baloh RW. Association of benign recurrent vertigo and migraine in 208 patients. Cephalalgia. (2009) 29:550–5. 10.1111/j.1468-2982.2008.01770.x 19170697 PMC2820365

[24] Lee H Sohn SI Jung DK Cho YW Lim JG Yi SD. Migraine and isolated recurrent vertigo of unknown cause. Neurol Res. (2002) 24:663–5. 10.1179/016164102101200726 12392202

[25] Moretti G Manzoni GC Caffarra P Parma M. “Benign recurrent vertigo” and its connection with migraine. Headache. (1980) 20:344–6. 10.1111/j.1526-4610.1980.hed2006344.x 7216756

[26] Von Brevern M Zeise D Neuhauser H Clarke AH Lempert T. Acute migrainous vertigo: clinical and oculographic findings. Brain. (2005) 128:365–74. 10.1093/brain/awh351 15601663

[27] Lempert T Von Brevern. M. Vestibular migraine. Neurol Clin. (2019) 37:695–706. 10.1016/j.ncl.2019.06.003 31563227

[28] Young AS Lechner C Bradshaw AP Macdougall HG Black DA Halmagyi GM. Capturing acute vertigo: a vestibular event monitor. Neurology. (2019) 92:e2743–53. 10.1212/WNL.0000000000007644 31092626

[29] Van Leeuwen RB Bruintjes TD. Recurrent vestibulopathy: natural course and prognostic factors. J Laryngol Otol. (2010) 124:19–22. 10.1017/S0022215109991009 19775488

[30] Teggi R Colombo B Albera R Asprella Libonati G Balzanelli C Batuecas Caletrio A. Clinical features, familial history, and migraine precursors in patients with definite vestibular migraine: the VM-phenotypes projects. Headache. (2018) 58:534–44. 10.1111/head.13240 29205326

[31] Teggi R Colombo B Albera R Asprella Libonati G Balzanelli C Batuecas Caletrio A. Clinical features of headache in patients with diagnosis of definite vestibular migraine: the VM-phenotypes projects. Front Neurol. (2018) 9:395. 10.3389/fneur.2018.00395 29922214 PMC5996089

[32] Neuhauser HK. The epidemiology of dizziness and vertigo. Handb Clin Neurol. (2016) 137:67–82. 10.1016/B978-0-444-63437-5.00005-4 27638063

[33] Becker-Bense S Wittmann C Dieterich M. Balanced sex distribution in patients with Menière's disease. J Neurol. (2019) 266:42–6. 10.1007/s00415-019-09301-4 30972498

[34] Dlugaiczyk J Habs M Dieterich M. Vestibular evoked myogenic potentials in vestibular migraine and Meniere's disease: cVEMPs make the difference. J Neurol. (2020) 267 (Suppl 1):169–80. 10.1016/B978-0-12-809324-5.23771-1 32494851 PMC7718204

[35] Kutlubaev MA Pyykko I Hardy TA Gürkov R. Menière's disease. Pract Neurol. (2020). 10.1136/practneurol-2020-002734. [Epub ahead of print]. 33249404

[36] Pyykk ö I Nakashima T Yoshida T Zou J Naganawa S. Meniere's disease: a reappraisal supported by a variable latency of symptoms and the MRI visualisation of endolymphatic hydrops. BMJ Open. (2013) 3:e001555. 10.1136/bmjopen-2012-001555 23418296 PMC3586172

[37] Van Der Lubbe M Vaidyanathan A Van Rompaey V Postma AA Bruintjes TD Kimenai DM. The “hype” of hydrops in classifying vestibular disorders: a narrative review. J Neurol. (2020) 267:197–211. 10.1007/s00415-020-10278-8 33201310 PMC7718205

[38] Paparella MM Mancini F. Vestibular Meniere's disease. Otolaryngol Head Neck Surg. (1985) 93:148–51. 10.1177/019459988509300203 3921902

[39] Lee SU Choi JY Kim HJ Kim JS. Recurrent spontaneous vertigo with interictal headshaking nystagmus. Neurology. (2018) 90:e2135–45. 10.1212/WNL.0000000000005689 29792303

[40] Bisdorff A Kattah J. Description of a new type of benign recurrent vertigo of central origin. Neurology. (2018) 90:1089–90. 10.1212/WNL.0000000000005683 29792304

[41] Requena T Espinosa-Sanchez JM Cabrera S Trinidad G Soto-Varela A Santos-Perez S. Familial clustering and genetic heterogeneity in Meniere's disease. Clin Genet. (2014) 85:245–52. 10.1111/cge.12150 23521103

[42] Paz-Tamayo A Perez-Carpena P Lopez-Escamez JA. Systematic review of prevalence studies and familial aggregation in vestibular migraine. Front Genet. (2020) 11:954. 10.3389/fgene.2020.00954 33110417 PMC7489493

