# Supplementary material for: Recurrent Vestibular Symptoms Not Otherwise Specified: Clinical Characteristics Compared With Vestibular Migraine and Menière's Disease
Source: Front Neurol. 2021 Jun 17;12:674092. doi: 10.3389/fneur.2021.674092 (PMC8248237; doi:10.3389/fneur.2021.674092)
Supplement: Supplementary Material 1 — “Vertigo PEVS” questionnaire (PEVS = prospective study on the phenotype of episodic vestibular syndromes). [file Data_Sheet_1.PDF]

VERTIGO PEVS

Prospective study on the phenotype of episodic vestibular syndromes

Participant ID: __ __ - __ __ __
AA - ###

Date: __ / __ / ____
DD / MM / YYYY

1. Socio-demographic:

Gender: ☐ Male ☐ Female

Date of birth: __ / __ / ____
DD / MM / YYYY

2. Duration of vestibular syndrome:

(!) ***Indicate number of months (could also be a fraction of a month).***

Duration: ____ , __ month(s)

3. Symptom quality according to Bárány Vestibular Symptoms grid.

(!) ***The Bárány vestibular symptoms grid is presented on the 4 next pages. The boxes on the last column represent specific symptoms.***
Only tick off current symptoms (those items which are not marked will be coded as absent).

Bárány Vestibular Symptoms grid (part 1/4)

- **1. (Internal) vertigo** is the sensation of self-motion (of head/body) when no self-motion is occurring or the sensation of distorted self-motion during an otherwise normal head movement.
  - **1. Spontaneous vertigo**
    - **1. Spinning** — **1.1.1** ☐
    - **2. Non-spinning** (rocking, swaying, etc.) — **1.1.2** ☐
  - **2. Triggered vertigo**
    - **1. Positional vertigo** is vertigo triggered by and occurring *after* a change of head position in space relative to gravity
      - **1. Transient** <1 minute
        - **1. Spinning** — **1.2.1.1.1** ☐
        - **2. Non-spinning** — **1.2.1.1.2** ☐
      - **2. Persistent** ≥1 minute
        - **1. Spinning** — **1.2.1.2.1** ☐
        - **2. Non-spinning** — **1.2.1.2.2** ☐
    - **2. Head-motion vertigo** vertigo occurring only *during* head motion (that is time-locked to the head movement)
      - **1. Spinning** — **1.2.2.1** ☐
      - **2. Non-spinning** — **1.2.2.2** ☐
    - **3. Visually-induced vertigo** is triggered by visual stimuli, including the relative motion of the visual surround associated with body movement
      - **1. Spinning** — **1.2.3.1** ☐
      - **2. Non-spinning** — **1.2.3.2** ☐
    - **4. Sound-induced vertigo**
      - **1. Spinning** — **1.2.4.1** ☐
      - **2. Non-spinning** — **1.2.4.2** ☐
    - **5. Valsalva-induced vertigo**
      - **1. Glottic**
        - **1. Spinning** — **1.2.5.1.1** ☐
        - **2. Non-spinning** — **1.2.5.1.2** ☐
      - **2. Nose pinch**
        - **1. Spinning** — **1.2.5.2.1** ☐
        - **2. Non-spinning** — **1.2.5.2.2** ☐
    - **6. Orthostatic vertigo** occurs at a change of body posture from lying to sitting or sitting to standing
      - **1. Spinning** — **1.2.6.1** ☐
      - **2. Non-spinning** — **1.2.6.2** ☐
    - **7. Other triggered vertigo**
      - **1. Spinning** — **1.2.7.1** ☐
      - **2. Non-spinning** — **1.2.7.2** ☐

Bárány Vestibular Symptoms grid (part 2/4)

- **2. Dizziness** is the sensation of disturbed or impaired spatial orientation without a false or distorted sense of motion
  - **1. Spontaneous dizziness** — **2.1** ☐
  - **2. Triggered dizziness**
    - **1. Positional dizziness** is dizziness triggered by and occurring *after* a change of head position in space relative to gravity
      - **1. Transient** <1 minute — **2.2.1.1** ☐
      - **2. Persistent** ≥1 minute — **2.2.1.2** ☐
    - **2. Head-motion dizziness** dizziness occurring only *during* head motion (that is time-locked to the head movement) — **2.2.2** ☐
    - **3. Visually-induced dizziness** is triggered by visual stimuli, including the relative motion of the visual surround associated with body movement — **2.2.3** ☐
    - **4. Sound-induced dizziness** — **2.2.4** ☐
    - **5. Valsalva-induced dizziness**
      - **1. Glottic** — **2.2.5.1** ☐
      - **2. Nose pinch** — **2.2.5.2** ☐
    - **6. Orthostatic dizziness** occurs at a change of body posture from lying to sitting or sitting to standing — **2.2.6** ☐
    - **7. Other triggered dizziness** — **2.2.7** ☐

Bárány Vestibular Symptoms grid (part 3/4)

3. Vestibulo-visual symptoms

- **1. External vertigo** is the false sensation that the visual surround is spinning or flowing — **3.1** ☐
- **2. Oscillopsia** is the false sensation that the visual surround is oscillating
  - **1. Head-movement dependent** — **3.2.1** ☐
  - **2. Occurs without head movements** — **3.2.2** ☐
- **3. Visual lag** is the sensation that the visual surround follows behind a head movement — **3.3** ☐
- **4. Visual tilt** is the false perception of the visual surround as oriented off the true vertical — **3.4** ☐
- **5. Movement-induced blur** is reduced visual acuity during or momentarily after a head movement — **3.5** ☐

Bárány Vestibular Symptoms grid (part 4/4)

- **4. Postural symptoms**
  - **1. Unsteadiness** is the feeling of being unstable while seated, standing, or walking — **4.1** ☐
  - **2. Directional pulsion** is the feeling of being unstable with a tendency to veer or fall in a particular direction
    - **1. Latero**
      - **1. Right** — **4.2.1.1** ☐
      - **2. Left** — **4.2.1.2** ☐
    - **2. Antero** — **4.2.2** ☐
    - **3. Retro** — **4.2.3** ☐
  - **3. Balance-associated near fall** — **4.3** ☐
  - **4. Balance-associated fall** — **4.4** ☐

Definitions for the purpose of this study:
A symptomatic episode (**attack**) is a distinct lapse of time during which symptoms are continuously present, during an attack moments with stronger intensity of symptoms may occur (**exacerbations**), e.g. in vestibular migraine an attack may last for hours and exacerbations during head motion or in certain visual surrounds. Periods where many attacks occur are **clusters** separated by periods of few or no attacks.

4. Attack frequency:

4.1. What is the frequency of attacks?

❗ **Choose only 1 answer.**

- ☐ Less than 1 time / year
- ☐ ≥ 1 / 12 months
- ☐ ≥ 1 / 6 months
- ☐ ≥ 1 / 3 months
- ☐ ≥ 1 / month
- ☐ ≥ 1 / week
- ☐ ≥ 1 / day
- ☐ Patient unable to answer question

4.2. Do attacks occur in clusters?

- ☐ No ➡ *If **NO**, go to question 4.4 (Residual symptoms between attacks).*
- ☐ Yes ➡ *If **YES**, continue.*
- ☐ Patient unable to answer question ➡ *Go to question 4.4.*

4.3. Usual duration of clusters:

❗ **Choose only 1 answer.**

- ☐ Weeks
- ☐ Months
- ☐ Patient unable to answer question

4.4. Are there residual symptoms between attacks?

- ☐ No ➡ *If **NO**, go to question 5 (Attack durations).*
- ☐ Yes ➡ *If **YES**, continue.*
- ☐ Patient unable to answer question ➡ *Go to question 5.*

4.5. Which symptoms?

(!) *More than 1 answer possible.*

- ☐ Spontaneous vertigo
- ☐ Head motion vertigo
- ☐ Visually induced vertigo
- ☐ Spontaneous dizziness
- ☐ Head motion dizziness
- ☐ Visually induced dizziness
- ☐ Unsteadiness
- ☐ Others
- ☐ Patient unable to answer question

4.6. Are the residual symptoms between attacks only during clusters?

(!) *Complete only if patient has clusters (answer to question 4.2 = Yes).*

- ☐ No
- ☐ Yes
- ☐ Patient unable to answer question

5. Attack durations:

5.1. Work out duration of core event, distinguish from "entire time to recovery to baseline": the core event is the time of presence of vestibular symptoms, this period may be followed by a period of fatigue, feeling unwell, etc., before full recovery to baseline.

(!) *More than 1 answer possible.*

- ☐ < 1 minute
- ☐ 1 - 5 minutes
- ☐ 6 - < 60 minutes
- ☐ 1 - 4 hours
- ☐ 5 - 24 hours
- ☐ Up to 3 days
- ☐ > 3 days
- ☐ Patient unable to answer question

5.2. Usual duration of recovery time:

(!) **Choose only 1 answer.**

- ☐ Immediate
- ☐ < 1 minute
- ☐ 1 - 5 minutes
- ☐ 6 - < 60 minutes
- ☐ 1 - 4 hours
- ☐ 5 - 24 hours
- ☐ Up to 3 days
- ☐ > 3 days
- ☐ Too variable to tell
- ☐ Patient unable to answer question

5.3. Are there distinctive exacerbations within an attack?

- ☐ No ➡ *If **NO**, go to question 6 (Intensity of symptoms).*
- ☐ Yes ➡ *If **YES**, continue.*
- ☐ Patient unable to answer question ➡ *Go to question 6.*

5.4. Duration of exacerbations:

(!) ***More than 1 answer possible.***

- ☐ < 1 minute
- ☐ 1 - 5 minutes
- ☐ 6 - < 60 minutes
- ☐ 1 - 4 hours
- ☐ > 4 hours
- ☐ Patient unable to answer question

5.5. Frequency of exacerbations:

(!) **Choose only 1 answer.**

- ☐ ≤ 1 / day
- ☐ 2 - 4 / day
- ☐ > 4 / day
- ☐ Patient unable to answer question

6. Intensity of symptoms:

6.1. How are most of your attacks?

! Choose only 1 answer.

- ☐ Mild (does not interfere in daily activities)
- ☐ Moderate (interferes with daily activities)
- ☐ Severe (daily activities are not possible)
- ☐ Patient unable to answer question

➡ *If **Mild**, **Moderate** or **Unable to answer**, continue.*

➡ *If **Severe**, go to question 7 (Accompanying symptoms).*

6.2. Do you have severe attacks?

- ☐ No
- ☐ Yes
- ☐ Patient unable to answer question

7. Accompanying symptoms of attacks:

Specify the frequency (mostly, sometimes, never) of each accompanying symptom.

Vision related	**Never**	**Sometimes**	**Mostly**
Photophobia	☐	☐	☐
Visual aura (define from IHS)	☐	☐	☐
Diplopia	☐	☐	☐

Hearing related	**Never**	**Sometimes**	**Mostly**
Phonophobia	☐	☐	☐
Tinnitus	☐	☐	☐
Fullness of ear	☐	☐	☐
Hearing loss	☐	☐	☐

Vegetative	**Never**	**Sometimes**	**Mostly**
Nausea	☐	☐	☐
Vomiting	☐	☐	☐
Palpitations	☐	☐	☐
Choking	☐	☐	☐

Emotional	**Never**	**Sometimes**	**Mostly**
Anxiety	☐	☐	☐

Headache?

☐ No ➡ *If **NO**, go to question 8 (Clinical diagnosis).*
☐ Yes ➡ *If **YES**, continue.*

Headache characteristics	**Never**	**Sometimes**	**Mostly**
Hemicranial	☐	☐	☐
Pulsating quality	☐	☐	☐
Worse on effort	☐	☐	☐
Moderate or severe intensity	☐	☐	☐

8. Clinical diagnosis:

! ***Only one answer possible.***

- ☐ Menière's disease clinically definite
- ☐ Definite vestibular migraine
- ☐ Probable vestibular migraine
- ☐ BPPV
- ☐ Vestibular paroxysmia [1]
- ☐ Definite vertebrobasilar Transitory Ischemic Attack (TIA) [2]
- ☐ Probable vertebrobasilar Transitory Ischemic Attack (TIA) [3]
- ☐ Panic attacks
- ☐ Recurrent vestibular symptoms NOS [4]

For the purpose of this study patients with more than one episodic vestibular diagnosis need to be excluded.

Definitions of clinical diagnosis:

[1] **Vestibular paroxysmia:**
Attacks of < 5 minutes, at least several per week for at least 3 months, and CBZ response (at least 50% reduction of attack frequency), exclusion of other causes.

[2] **Definite vertebrobasilar Transitory Ischemic Attack (TIA):**
Patients having at least one event not longer than 6 months before with vestibular symptoms, with or without other posterior fossa symptoms, lasting from 5 minutes to 24 hours, and an index event (acute event with vestibular symptoms) with at least 1 of the following 3 criteria fulfilled:

1. clinical diagnosis of a posterior fossa stroke;
2. recent ischemic stroke on imaging in posterior circulation;
3. proof of significant vascular stenosis in posterior circulation.

And no better explanation for vestibular symptoms.

[3] **Probable vertebrobasilar Transitory Ischemic Attack (TIA):**
Patients having at least two events not starting longer than 6 months before with vestibular symptoms, with or without other posterior fossa symptoms, lasting from 5 minutes to 24 hours and points 1-3 fulfilled:

1. age > 60;
2. at least 2 cardio-vascular risk factors:
  - **a.** diabetes;
  - **b.** arterial hypertension;
  - **c.** hyperlipidemia;
  - **d.** smoking;
  - **e.** previous stroke / myocardial infarction.

3. no better explanation for vestibular symptoms.

[4] **Recurrent vestibular symptoms NOS (not otherwise specified):**
Recurrent episodes of vestibular symptoms lasting for at least 5 minutes and not fulfilling the criteria of Menière's disease, vestibular migraine or probable/definite TIA. Other specified diagnoses like perilymphatic fistula or episodic ataxia are excluded.
